# Merging of the photocatalysis and copper catalysis in metal–organic frameworks for oxidative C–C bond formation†
†Electronic supplementary information (ESI) available. CCDC 997028 and 997029. For ESI and crystallographic data in CIF or other electronic format see DOI: 10.1039/c4sc02362e



**DOI:** 10.1039/c4sc02362e

**Published:** 2014-10-30

**Authors:** Dongying Shi, Cheng He, Bo Qi, Cong Chen, Jingyang Niu, Chunying Duan

**Affiliations:** a State Key Laboratory of Fine Chemicals , Dalian University of Technology , Dalian , 116024 , P. R. China . Email: cyduan@dlut.edu.cn; b College of Chemistry and Chemical Engineering , Henan University , Kaifeng , 475004 , P. R. China

## Abstract

The direct formation of new C–C bonds through photocatalytic oxidative coupling from low reactive sp^3^ C–H bonds using environmentally benign and cheap oxygen as oxidant is an important area in sustainable chemistry. By incorporating the photoredox catalyst [SiW_11_O_39_Ru(H_2_O)]^5–^ into the pores of Cu-based metal–organic frameworks, a new approach for merging Cu-catalysis/Ru-photocatalysis within one single MOF was achieved. The direct Cu^II^–O–W(Ru) bridges made the two metal catalyses being synergetic, enabling the application on the catalysis of the oxidative coupling C–C bond formation from acetophenones and *N*-phenyl-tetrahydroisoquinoline with excellent conversion and size-selectivity. The method takes advantage of visible light photoredox catalysis to generate iminium ion intermediate from *N*-phenyl-tetrahydroisoquinoline under mild conditions and the easy combination with Cu-catalyzed activation of nucleophiles. Control catalytic experiments using similar Cu-based sheets but with the photoredox catalytic anions embedded was also investigated for comparison.

## Abstract 2

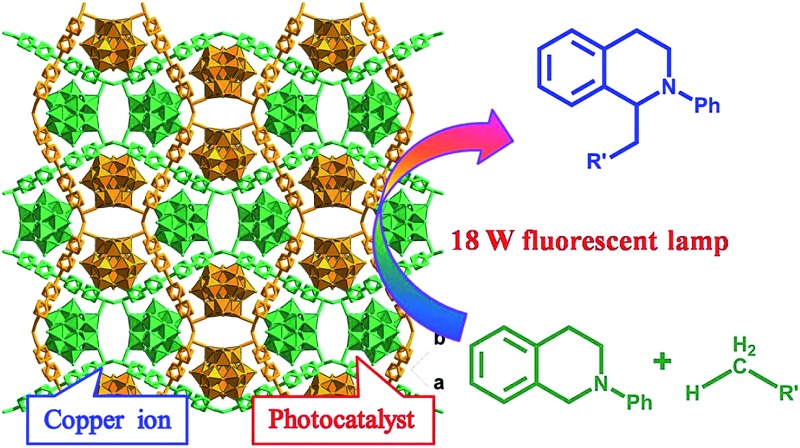
A new approach to merge Cu-catalysis/Ru-photocatalysis within one single MOF was achieved by incorporating [SiW_11_O_39_Ru(H_2_O)]^5–^ into Cu–**BPY** MOFs.

## Introduction

The direct formation of new C–C bonds through oxidative coupling reactions from the lower active sp^3^ C–H bonds using oxygen as oxidant is an important area in sustainable chemistry.^1^ Among the reported promising examples, the oxidative activation of the C–H bonds adjacent to nitrogen atom in tertiary amines represents a powerful strategy, giving valuable, highly reactive iminium ion intermediates for further functionalization.^2^ Recent investigations also revealed that the visible light photoredox catalysis was a promising approach to such reaction sequences^3^ with respect to the development of new sustainable and green synthetic methods. It was also postulated that the combination of the photocatalysis and the metal catalysis within a dual catalytic transformation is attractive to circumvent the potential side reactions relative to the highly active intermediates that exist in the photocatalysis.^4^ The hurdles that need to be overcome include the careful adaptation and the fine tuning of the reaction rates of the two catalytic cycles,^5^ beside the appropriate choice of the metal catalysis and photocatalysis.^6^

Metal–organic frameworks are hybrid solids with infinite network structures built from organic bridging ligands and inorganic connecting nodes. Besides the potential applications in many diverse areas,^7^ MOFs are ideally suited for catalytic conversions, since they can impose size and shape selective restriction through readily fine-tuned channels or pores,^8^ providing precise knowledge about the pore structure, the nature and distribution of catalytically active sites.^9^ In comparison to the heterogeneous catalytic systems that have been examined earlier, the design flexibility and framework tunability resulting from the huge variations of metal nodes and organic linkers allow the introduction of more than two independent catalyses in one single MOF.^10^ The combination of photocatalysis with the metal ions or organocatalysis was expected to be a promising approach to create synergistic catalysts.^11^

**Scheme 1:**
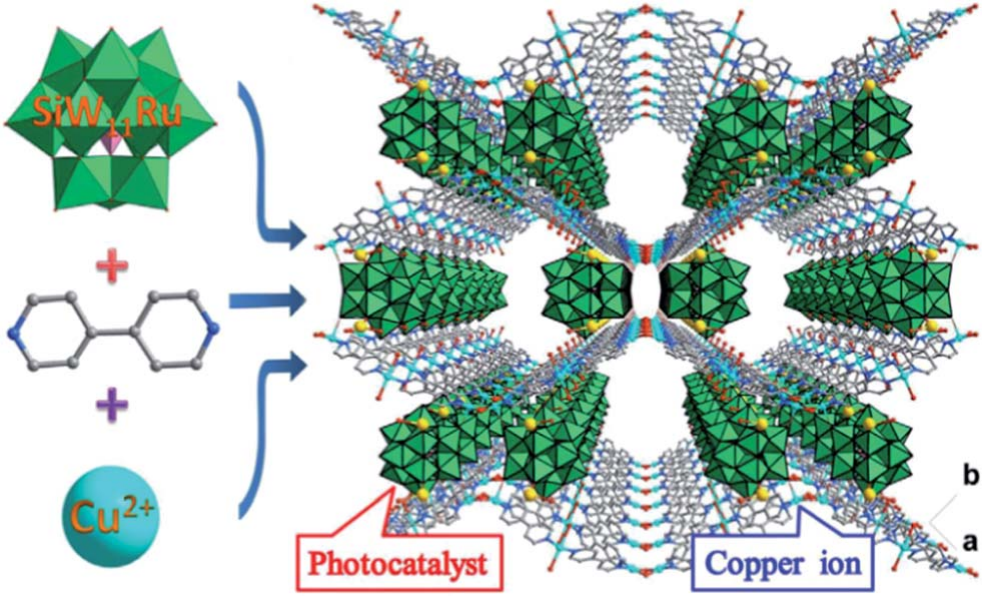
Synthetic procedure for the 3D CR–**BPY**1 MOF that is composed of wavy-like Cu–**BPY** sheets and [SiW_11_O_40_Ru]^7–^ anions showing the combination of the dual catalytic units and channels for chemical transformations.

By incorporating a ruthenium(iii) substituted polyoxometalate [SiW_11_O_39_Ru(H_2_O)]^5–^ within the pores of copper(ii)-bipyridine MOFs, herein, we reported a new approach to merge the visible light photocatalytic aerobic oxidation and copper(ii) catalytic coupling reaction within one MOF (Scheme 1). We envisioned that the ruthenium-containing fragments possibly worked as oxidative photocatalyst to generate the iminium ion from *N*-phenyl-tetrahydroisoquinolines,^12^ whereas the Cu-based MOF potentially activated the nucleophiles, as it was shown in the oxidative C–C bond coupling.^13^

## Results and discussion

### Synthesis and characterizations of CR–**BPY**1

Solvothermal reaction of 4,4′-bipyridine (**BPY**), Cu(NO_3_)_2_·3H_2_O and K_5_[SiW_11_O_39_Ru(H_2_O)]·10H_2_O gave CR–**BPY**1 in a yield of 52%. Elemental analyses and powder X-ray analysis indicated the pure phase of its bulk sample. Single-crystal structural analysis revealed that CR–**BPY**1 crystallized in a space group *P*42_1_ *m*. Two crystallographically independent copper(ii) ions are connected by **BPY** ligands and μ_2_-water bridges alternatively to produce 2D wavy-like Cu–**BPY** sheets (Fig. S5, ESI†). The Cu(2) atom adopted a six-coordinate octahedral geometry with four nitrogen atoms from four **BPY** ligands positioned in the equatorial plane and two water molecules occupied the axial positions. The Cu(1) atom displayed a five-coordinate square pyramidal geometry with two μ_2_-water groups and two nitrogen atoms of **BPY** ligands positioned in the basal plane, and a terminal oxygen atom of the depronated [SiW_11_O_39_Ru(H_2_O)]^5–^ polyoxoanion occupied the vertex position. The ruthenium atom disordered in the twelve equivalent positions within a depronated [SiW_11_O_39_Ru(H_2_O)]^5–^.^14^ The availability of vacant d-orbitals on the metal atoms adjacent to the heteroatom allows the polyoxometalate matrix to function as a π-acceptor ligand.^15^

**Fig. 1:**
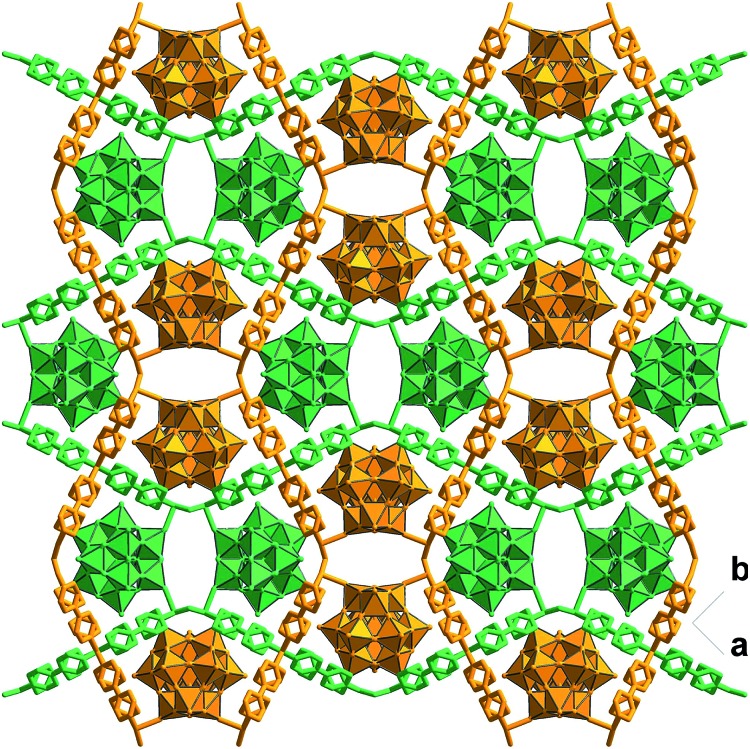
Crystal structure of CR–**BPY**1 with the 3D framework generated by the covalently linking of the wavy-like 2D sheets (drawn in stick-ball model) and the [SiW_11_O_39_Ru(H_2_O)]^5–^ (drawn in polyhedron) by Cu^II^–O–W(Ru) bridges, showing the interpenetration of two symmetric frameworks.

While these copper atoms were connected by the **BPY** ligands to form two-dimension square grid at first, adjacent sheets were connected together using the deprotonated [SiW_11_O_39_Ru(H_2_O)]^5–^ polyoxoanion by Cu^II^–O–W(Ru) bridges to generate a 3D framework. Two symmetric-related frameworks further interpenetrated each other perpendicularly to consolidate the robust structure (Fig. 1), in which the opening of the pores was reduced to 10.0 Å × 5.3 Å. To the best of our knowledge, CR–**BPY**1 represents the first example of MOFs which are comprised of ruthenium substituted polyoxometalate [SiW_11_O_39_Ru(H_2_O)]^5–^. As the noble metal substituted polyoxometalates exhibited excellent photoreactivity in various catalytic oxidation processes of organic substrates,^16^ such kinds of MOFs potentially allow the combination of photocatalysis and MOF-based heterogeneous catalysis to achieve synthetically useful organic transformations. Moreover, the directly bridging of the copper and ruthenium by Cu^II^–O–W(Ru) provided a promising way to achieve the synergistic catalysis between photocatalyst and metal catalyst.

**Fig. 2:**
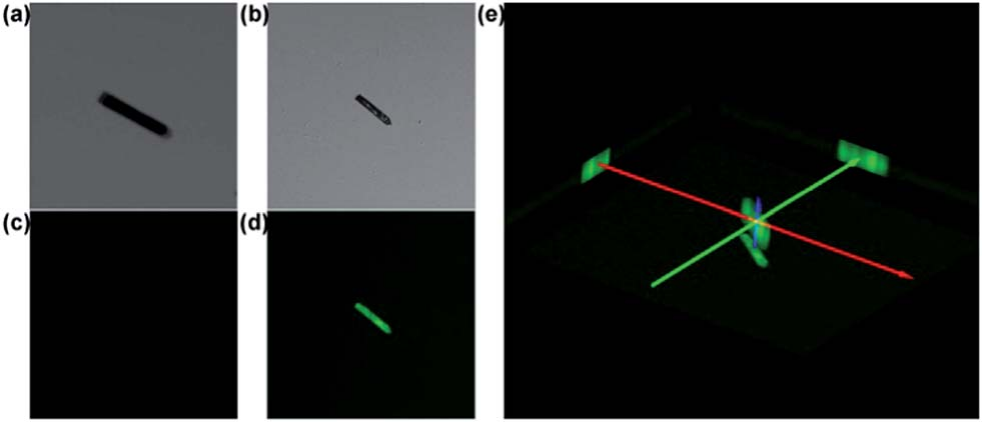
Confocal images of empty (a and c) and soaked (b and d) 2′,7′-dichlorofluorescein dye. Brightfield images (a and b) and confocal images (c and d) detected at *λ*_em_ = 510–610 nm, exited by *λ*_ex_ = 488 nm through a 405/488 nm filter. (e) The 3D reconstruction of the soaked 2′,7′-dichlorofluorescein dye (b). Three images at the end of axes in (e) exemplify the *X*, *Y* and *Z*-axis projections of the soaked dye.

Confocal fluorescence microscopy has attracted much attention in biological imaging. It may provide a way to analyse relatively thick porous materials, because it offers the advantage of increased penetration depth (>500 mm).^17^ The assessment of guest-accessible volume in MOFs can be reliably done by using confocal fluorescence microscopy with a tool-box of dyes with a wide range of sizes. It would be applicable to any porous materials, whose single-crystal structures are not available, or non-crystalline materials.^18^ Dye uptake investigation was carried out by soaking CR–**BPY**1 in a methanol solution of 2′,7′-dichlorofluorescein. It gave the quantum uptake equivalent to 5% of the MOF weight (Fig. S11, ESI†).^19^ The confocal laser scanning microscopy exhibited strong green fluorescence (*λ*_ex_ = 488 nm) assignable to the emission of the fluorescein dye (Fig. 2), confirming the successful uptake of the dye molecules inside the crystals of the MOF.^20^ Furthermore, the rather uniform distribution of the dye molecules throughout the crystal suggested that the dyes penetrated deeply into the crystal rather than staying on the external surface. Without guest water molecules, the effective free volume of CR–**BPY**1 was estimated to be 29.0% by PLATON software.^21^

**Fig. 3:**
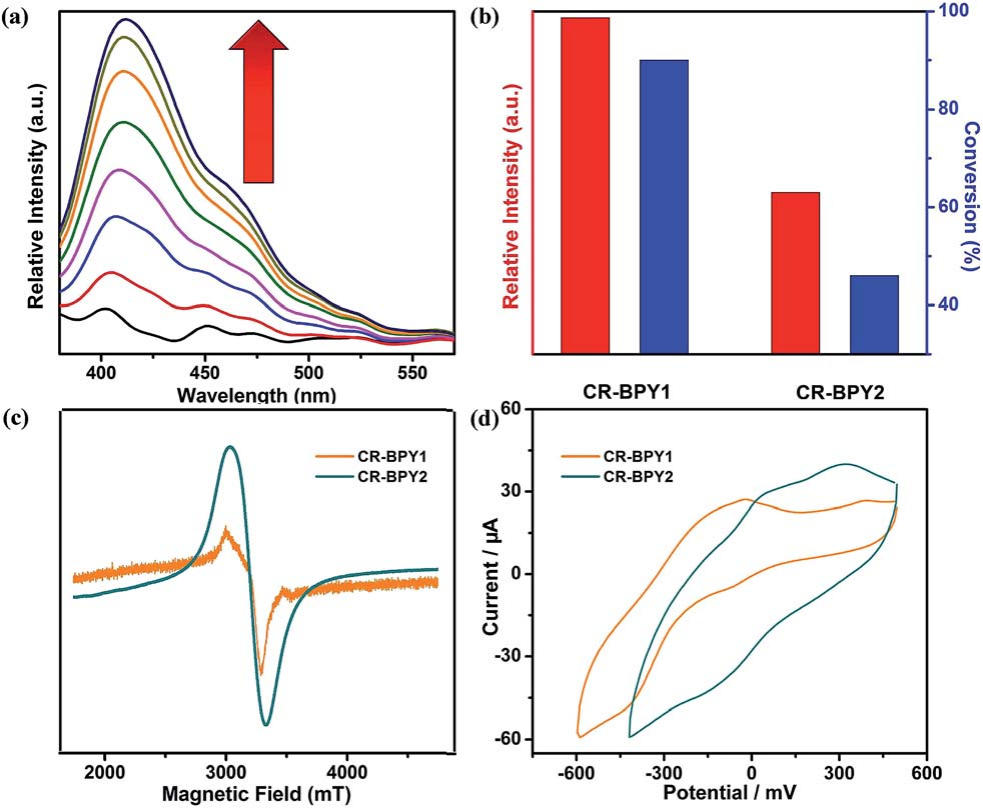
(a) Family of emission spectra of CR–**BPY**1 (0.1% in weight) in CH_2_Cl_2_ suspension upon addition of *N*-phenyl-tetrahydroisoquinoline up to 0.50 mM, excitation at 362 nm. (b) The luminescence intensities of CR–**BPY**1 and CR–**BPY**2 upon addition of the same amount of *N*-phenyl-tetrahydroisoquinoline up to 0.50 mM at room temperature in CH_2_Cl_2_; the catalytic activities of CR–**BPY**1 and CR–**BPY**2 using *N*-phenyl-tetrahydroisoquinoline and nitromethane as the coupling partners. (c) EPR spectra of CR–**BPY**1 (*g* = 2.1438) and CR–**BPY**2 (*g* = 2.1320) in solid state at 77 K, respectively. (d) Solid state cyclic voltammetry of CR–**BPY**1 and CR–**BPY**2, respectively, scan rate: 50 mV s^–1^.

CR–**BPY**1 exhibited an absorption band centered at 398 nm in the solid state UV-vis absorption spectrum (Fig. S1, ESI†), assignable to the transitions of [SiW_11_O_39_Ru(H_2_O)]^5–^.^22^ Upon excitation at this band, CR–**BPY**1 did not exhibit any obvious emission, however, progressive addition of the *N*-phenyl-tetrahydroisoquinoline into the dichloromethane suspension of CR–**BPY**1 up to 0.50 mM caused the appearance of the Ru^II^-relative emission band at about 422 nm (Fig. 3a).^23^ The results suggested that CR–**BPY**1 oxidized *N*-phenyl-tetrahydroiso-quinoline to form the Ru^II^ species and the iminium intermediate.^24^ Electrospray ionization mass spectrometry of the CH_2_Cl_2_ suspension containing *N*-phenyl-tetrahydroisoquinoline and CR–**BPY**1 after 3 hours light irradiation exhibited an intense peak at *m*/*z* = 208. This peak was assignable to the relative imine ion, confirming that CR–**BPY**1 oxidized *N*-phenyl-tetrahydroisoquinoline to form the Ru^II^ species and the iminium intermediate (Fig. S13, ESI†). The electron paramagnetic resonance (EPR) of CR–**BPY**1 exhibited the characteristic signal of Cu^II^ with *g* = 2.14 (Fig. 3c). Solid state electrochemical measurements (Fig. 3d) exhibited a broad redox band centred at –186 mV (*vs.* SCE) relative to the overlap of the Cu^II^/Cu^I^ and Ru^III^/Ru^II^ redox couples. The potentials were comparable to these Cu^II^ and Ru^III^-containing catalysts,^25^ and enabled CR–**BPY**1 to prompt the oxidative coupling of *N*-phenyl-tetrahydroisoquinoline with nucleophiles under light.^26^ It seems that CR–**BPY**1 adsorbed the *N*-phenyl-tetrahydroisoquinoline in its pores and activated the substrate to form the iminium intermediate.

### Catalysis details of CR–**BPY**1

The catalysis was examined initially using *N*-phenyl-tetrahydroisoquinoline and nitromethane as the coupling partners, along with a common fluorescent lamp (18 W) as the light source. The resulting reaction gave a yield of 90% after 24 hours irradiation. The removal of CR–**BPY**1 by filtration after 18 hours shut down the reaction, and the filtrate afforded only 12% additional conversion for another 18 hours at the same reaction conditions. The observation suggested that CR–**BPY**1 was a true heterogeneous catalyst.^27^ Solids of CR–**BPY**1 could be isolated from the reaction suspension by simple filtration alone and reused at least three times with moderate loss of activity (from 90% to 82% of yield after three cycles). The index of XRD patterns of CR–**BPY**1 filtrated off from the reaction mixture suggested the maintenance of the crystallinity (Fig. S14, ESI†). With the size of the microcrystals reduced to 2 μm by grinding CR–**BPY**1 crystals for 20 min, the time of the reaction giving the same conversion to that of the as-synthesized materials was reduced by about 10% (Fig. S15, ESI†). It seems that the MOF-based particles having well-defined size were really helpful for the catalytic reactions, but the size of the crystals did not dominate the catalysis directly.

Control experiments for the C–C coupling reaction of *N*-phenyl-tetrahydroisoquinoline and nitromethane were carried out and summarized in Table 1. Almost no conversion was observed when the reaction was conducted in the dark (entry 7), while a very slow background reaction was observed in the absence of catalyst (entry 6), which demonstrated that both the light and the photocatalyst are required for efficient conversion to the coupling products. In addition, using the same equiv. of copper(ii) salts or/and K_5_[SiW_11_O_39_Ru(H_2_O)] as catalysts, respectively gives conversions of 39%, 25% and 42% in homogeneous fashion (entry 3–5). These results suggested that the direct connection of copper(ii) ions to [SiW_11_O_40_Ru]^7–^ anions not only enabled the dual catalysts to individually activate *N*-phenyl-tetrahydroisoquinoline and nitromethane, but also enforced the proximity between the potential intermediates *i.e.* the iminium ion and nucleophile, avoiding the unwanted side reactions or reverse reactions.^28^

**Table 1:** Control experiments for the C–C coupling reaction of *N*-phenyl-tetrahydroisoquinoline and nitromethane

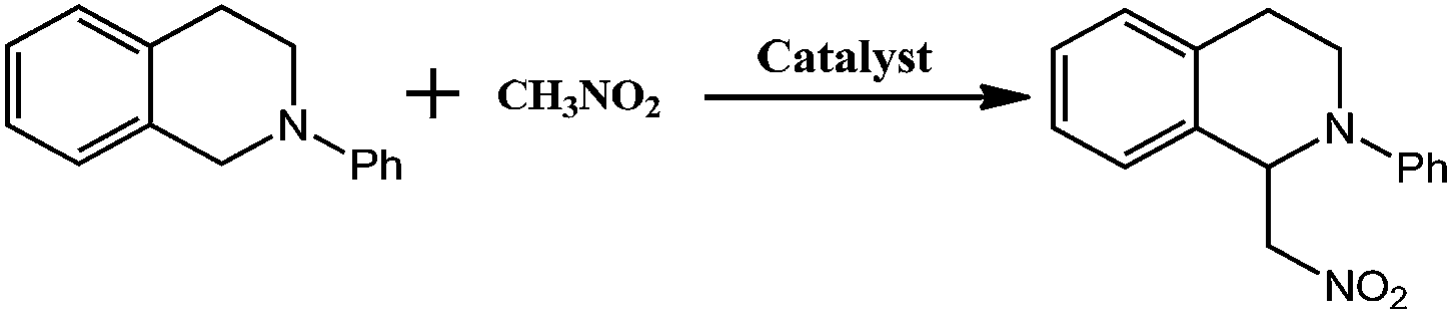		
Entry	Catalysts^*a*^	Conversion^*b*^ (%)
1	CR–**BPY**1	90
2	CR–**BPY**2	46
3	Cu(NO_3_)_2_·3H_2_O	39
4	K_5_[SiW_11_O_39_Ru(H_2_O)]	25
5	K_5_[SiW_11_O_39_Ru(H_2_O)], Cu(NO_3_)_2_·3H_2_O	42
6	No catalyst	20
7	CR–**BPY**1, no light	<10

^*a*^Reaction conditions: *N*-phenyl-tetrahydroisoquinoline (0.25 mmol), 1 mol% catalyst, 2.0 mL nitromethane, 18 W fluorescent lamp at room temperature.

^*b*^The conversions after 24 hour irradiation were determined by ^1^H NMR of crude products.

**Fig. 4:**
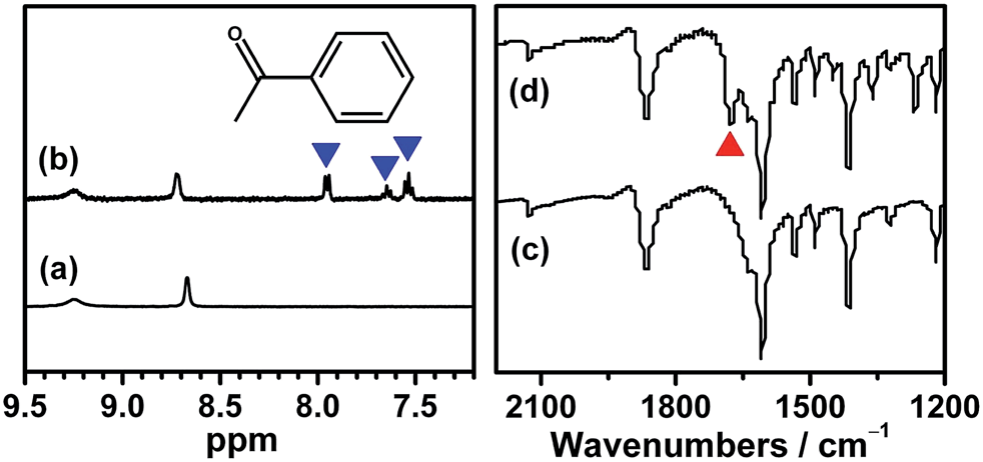
^1^H NMR in DCl/DMSO and solid state IR spectra of the desolvated CR–**BPY**1 (a) and (c), respectively, and of the desolvated CR–**BPY**1 impregnated in a dichloromethane solution of acetophenone (b) and (d), respectively, showing the absorbency and activation of substrate in the MOF. The blue and red triangle represented the signals of acetophenone in the NMR and IR spectra, respectively.

Although several examples of photocatalysts and metal copper catalysts have been reported to prompt the oxidative coupling C–C bond formation, CR–**BPY**1 represents a new example of a heterogeneous bimetal catalyst that merges the copper catalyst and the ruthenium(iii) substituted polyoxometalate catalyst within one single material. The high modularity of the systems allows an easy adaptation of the concept of dual catalysis, and represents an example of combining dual catalysis to achieve synthetically useful organic transformation. In this case, our catalytic systems were extended with other pronucleophiles, *i.e.* substituted acetophenones. As shown in Fig. 4, ^1^H NMR of desolvated CR–**BPY**1 solids immersed in a dichloromethane solution of acetophenone exhibited that CR–**BPY**1 could adsorb about 2 equiv. of acetophenone per copper(ii) moiety. IR spectrum of CR–**BPY**1 impregnated with a dichloromethane solution of acetophenone revealed a C=O stretching vibration at 1679 cm^–1^. The red shift from 1685 cm^–1^ (free acetophenone) suggested the adsorption and the activation of the acetophenone in the channels of the MOFs. It is hypothesized that the interactions between the copper ions in CR–**BPY**1 and the C=O groups of acetophenone possibly gave an active nucleophile.^29^

The reactions were carried out in the presence of a common used secondary amine, l-proline, as an organic co-catalyst to activate the ketones.^30^ In the case of the acetophenone as reactant with a fluorescent lamp (18 W) as the light source; the catalytic reaction gave a yield of 72%. Control experiments demonstrated that the use of K_5_[SiW_11_O_39_Ru(H_2_O)] or copper(ii) salts as catalysts, only gave less than 25% of the conversions, respectively. The results indicated the significant contribution of cooperative effects of the individual parts within one single MOF. From the mechanistic point of view, the ruthenium(iii) of the polyoxometalate [SiW_11_O_39_Ru(H_2_O)]^5–^ interacted with *N*-phenyl-tetrahydroisoquinoline to form iminium ions, whereas the copper atoms coordinated to the acetophenones weakly to form the enol intermediate that worked as active nucleophile for the oxidative coupling C–C bond formation. At the same time, the presence of copper ions could enhance the activation of *N*-phenyl-tetrahydroisoquinoline, benefiting the synergistic catalysis between photocatalyst and metal catalyst. Importantly, in contrast to the smooth reactions of substrates **1–3**, the C–C coupling reaction in the presence of bulky ketone (1-(3′,5′-di-*tert*-butyl [1,1′-biphenyl]-4-yl)-ethanone) **4**, gave less than 10% conversion under the same reaction conditions (Table 2, entry 4). The negligible adsorption by immersing CR–**BPY**1 into a dioxane solution of substrate **4**, coupled with the fact that the size of substrate **4** was larger than that of the channels,^31^ revealed that **4** was too large to be adsorbed in the channels. Furthermore, it is suggested that the synergistic catalytic coupling reaction indeed occurred in the channels of the MOF, not on the external surface.

**Table 2:** Photocatalytic oxidative C–C coupling reactions of *N*-phenyl-tetrahydroisoquinoline and substituted acetophenones^*a*^

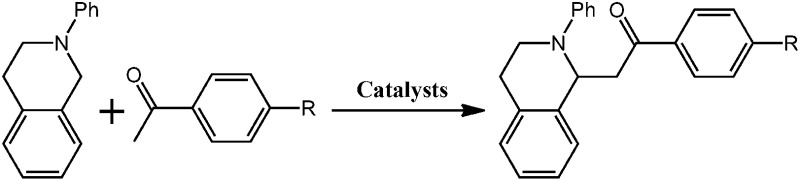			
Entry	Substrate	Molecular size	Conversion^*b*^ (%)
1	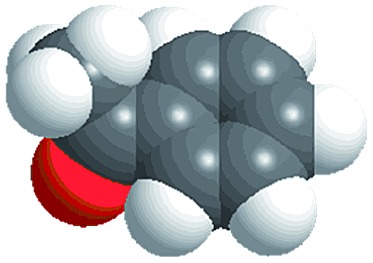	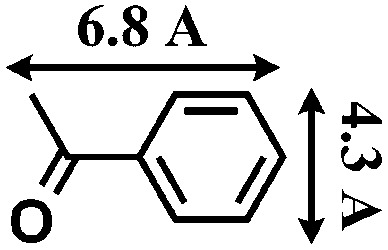	72
2	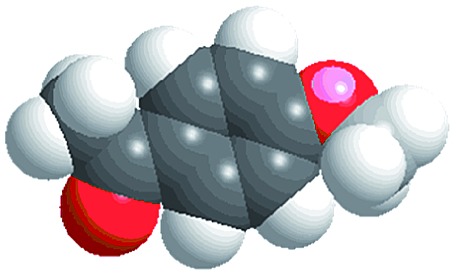	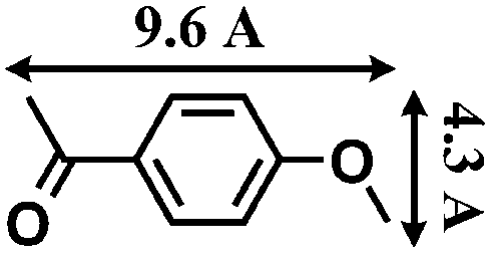	68
3	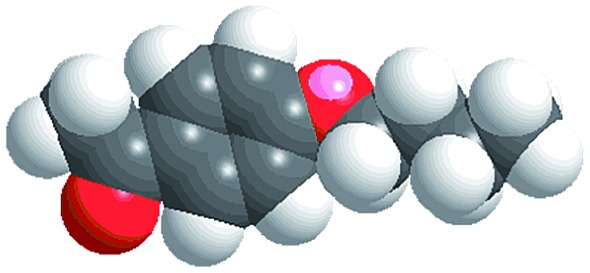	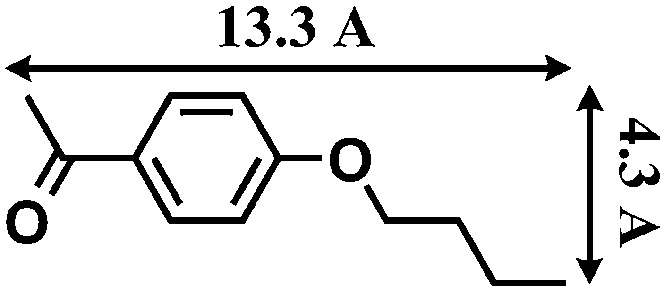	59
4	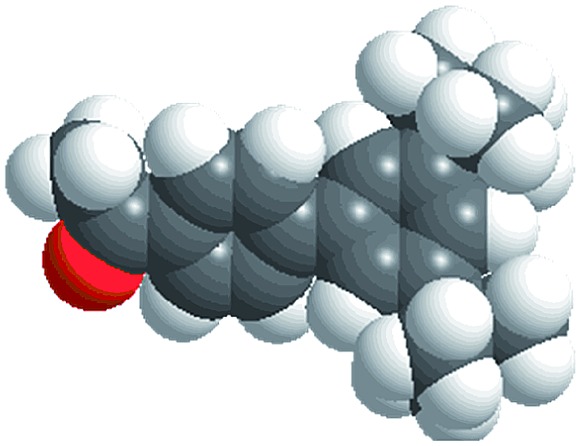	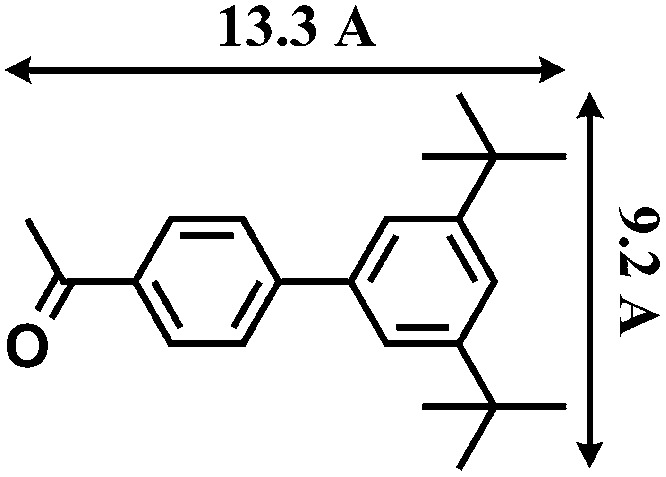	<10

^*a*^Reaction conditions: *N*-phenyl-tetrahydroisoquinoline (0.25 mmol), 1 mol% catalyst, 18 W fluorescent lamp, 20 mol% l-proline in 2.0 mL of 1,4-dioxane.

^*b*^The conversions were determined by ^1^H NMR spectroscopy of crude products.

### Synthesis and catalytic characterizations of CR–**BPY**2

**Fig. 5:**
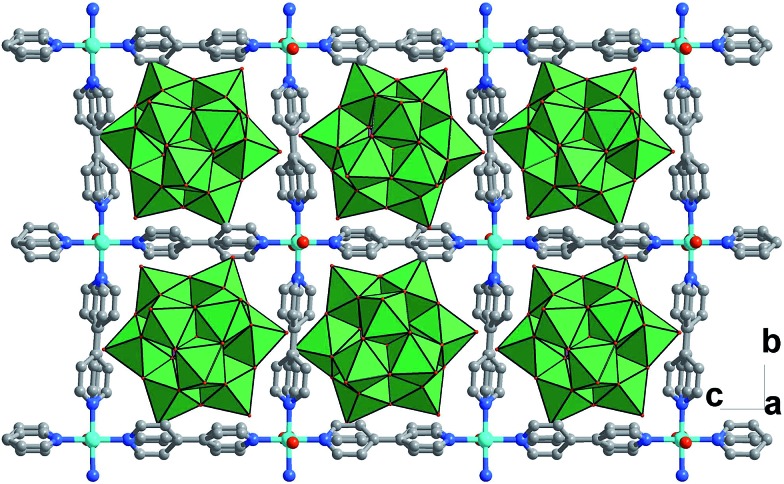
Crystal structure of CR–**BPY**2 showing the stacking pattern of the grid-like sheets with embedded [SiW_11_O_39_Ru(H_2_O)]^5–^. The atoms of copper, tungsten, carbon, nitrogen and oxygen were drawn in cyan, green, gray, dark blue and red, respectively.

To further investigate the synergistic interactions between the inorganic copper and [SiW_11_O_39_Ru(H_2_O)]^5–^ anion, a reference compound CR–**BPY**2 was assembled using the same starting components but different synthetic conditions (hierarchical diffusion). CR–**BPY**2 was synthesized by a diffusion method in a test tube by laying a solution of 4,4′-bipyridine in acetonitrile onto the solution of K_5_[SiW_11_O_39_Ru(H_2_O)]·10H_2_O and Cu(NO_3_)_2_·3H_2_O in water for several days in a yield of 59%. Elemental analyses and powder X-ray analysis indicated the pure phase of its bulk sample. Single-crystal structural analysis revealed that CR–**BPY**2 crystallized in the orthorhombic lattice with a space group *Pccn*. Two crystallographically independent copper(ii) ions connected four **BPY** bridges alternatively to produce a 2D sheet (Fig. 5), which were further stacked paralleled along the crystallographic *a* axis to form the 3D structure with embedded [SiW_11_O_39_Ru(H_2_O)]^5–^ (Fig. S8, ESI†). The copper(ii) ions resided in an octahedral geometries with the equatorial plane which was defined by four nitrogen atoms of **BPY** ligands, and the axial positions were occupied by two water molecules (Fig. S7, ESI†). Without guest water molecules, the effective free volume of CR–**BPY**2 was also estimated to be 33.9% by PLATON software, which is quite larger than that of CR–**BPY**1. These results suggested that the pore of CR–**BPY**2 is larger enough to adsorb the substrates. Since [SiW_11_O_39_Ru(H_2_O)]^5–^ polyoxoanions were embedded in the channels, it is thus an excellent reference for investigating the catalytic activity on the same coupling reaction.

CR–**BPY**2 also exhibited an absorption band centered at 398 nm in the solid state UV-vis absorption spectrum. Upon excitation at this band, CR–**BPY**2 did not exhibit obvious emission, however, progressive addition of the *N*-phenyl-tetrahydroisoquinoline into the dichloromethane suspension of CR–**BPY**2 up to 0.50 mM caused the appearance of the Ru^II^-relative emission band at about 422 nm, suggesting that CR–**BPY**2 oxidized *N*-phenyl-tetrahydroisoquinoline to form the Ru^II^ species. The EPR of CR–**BPY**2 exhibited the characteristic signal of Cu^II^ (*g* = 2.13).^32^ The sharper peak shape compared to that of CR–**BPY**1 might be one of the indicator of isolated Cu^II^ ions in CR–**BPY**2. No metal–metal interactions were found corresponding to the Cu^II^ ions in CR–**BPY**2. Solid state electrochemical measurements exhibited two redox peaks corresponding to the Cu^II^/Cu^I^ and Ru^III^/Ru^II^ redox couples, with the redox potential calculated at 75 mV and 84 mV (*vs.* SCE). The potentials enabled CR–**BPY**2 to prompt the oxidative coupling of *N*-phenyl-tetrahydroisoquinoline with nucleophiles under light. However, the separated redox peaks also suggested that these Cu^I^ and Ru^III^ ions did not interacted directly. It seems that CR–**BPY**2 adsorbed the *N*-phenyl-tetrahydroisoquinoline in its pores and was a convincing reference to investigate the synergistic action between Cu^II^–O–W(Ru) bimetal of CR–**BPY**1.

The catalytic activities of CR–**BPY**2 in the C–C coupling reactions were examined under the same conditions using nitromethane and *N*-phenyl-tetrahydroisoquinoline as the reactants. About 1 mol% loading amount of the catalyst gave rise to a 46% conversion, which was superior to the case when copper(ii) salts and the K_5_[SiW_11_O_39_Ru(H_2_O)] were employed as catalysts, indicating the significance of the two constitute parts for CR–**BPY**2 as a photocatalyst. However, the catalytic activities of CR–**BPY**2 were significantly weaker than that of CR–**BPY**1 (Fig. 3b). It should be concluded that the direct bridging of the copper and ruthenium by Cu^II^–O–W(Ru) provided a promising way to achieve the synergistic catalysis between photocatalyst and metal catalyst, and the high reaction efficiency in the reactions was dominated by the spacious environment of the channels, like those of CR–**BPY**1.

## Conclusions

In a summary, we reported the new example of copper MOFs containing the ruthenium substituted polyoxometalate with the aim of merging the synergistic Cu-catalysis/Ru-photocatalysis in a single MOF. CR–**BPY**1 exhibited perpendicularly inter-penetrated structure and the catalytic sites positioned in the robust pores of MOFs. Luminescence titration and IR spectra of the MOF-based material revealed the adsorbance and activation of *N*-phenyl-tetrahydroisoquinoline and acetophenone, by the ruthenium center and copper ions, respectively. The direct connection of copper(ii) ions to [SiW_11_O_40_Ru(H_2_O)]^5–^ not only provided the possibility of the dual catalysts to individually activate the substrates, but also enforced the proximity between the intermediates, avoiding the unwanted side reactions or reverse reactions. CR–**BPY**1 exhibited high activity for the photocatalytic oxidative coupling C–C bond formation with excellent size-selectivity, suggesting the catalytic reactions occurred in the channels of the MOF, and not on the external surface.

## Experimental section

### General methods and materials

All chemicals were of reagent grade quality obtained from commercial sources and used without further purification. ^1^H NMR was measured on a Varian INOVA-400 spectrometer with chemical shifts reported as ppm (in DMSO-d_6_ or CDCl_3_, TMS as internal standard). The elemental analyses (EA) of C, H and N were performed on a Vario EL III elemental analyzer. Inductively coupled plasma (ICP) analyses were performed on a NexION 300D spectrometer. The powder XRD diffractograms were obtained on a Riguku D/MAX-2400 X-ray diffractometer with Cu sealed tube (*λ* = 1.54178 Å). IR spectra were recorded as KBr pellets on a NEXUS instrument. Solid UV-vis spectra were recorded on a U-4100 spectrometer. Liquid UV-vis spectra were performed on a TU-1900 spectrophotometer. Solid fluorescent spectra were measured on a JASCO FP-6500 instrument. The excitation and emission slits were both 3 nm wide. Solid state cyclic voltammograms were measured on a Zahner PP211 instrument by using a carbon paste working electrode. Thermogravimetric analyses (TGA) were carried out at a ramp rate of 5 °C min^–1^ in nitrogen flow with a SDTQ600 instrument. Electron paramagnetic resonance (EPR) spectra were recorded on a EMX-10/12 spectrometer. Scanning electron microscopy (SEM) images were taken with a NOVA NanoSEM 450 microscope. All confocal laser scanning microscopy (CLSM) micrographs were collected by Olympus Fluoview FV1000. Products were purified by flash column chromatography on 200–300 mesh silica gel SiO_2_.

### Synthesis of CR–**BPY**1

K_5_[SiW_11_O_39_Ru(H_2_O)]·10H_2_O^33^ were prepared according to the literature methods and characterized by IR spectroscopy and UV-vis spectroscopy, respectively. A mixture of K_5_[SiW_11_O_39_Ru(H_2_O)]·10H_2_O (70.0 mg, 0.02 mmol), 4,4′-bipyridine (19.3 mg, 0.12 mmol) and Cu(NO_3_)_2_·3H_2_O (30.4 mg, 0.12 mmol) in mixed water (5.0 mL) and acetonitrile (3.0 mL) was stirred and its pH value was adjusted to 4.4 with 1 mol L^–1^ HAc. The resulting suspension was sealed in a 25 mL Teflon-lined reactor and kept at 120 °C for three days. After cooling the autoclave to room temperature, brownish black prismatic single crystals were separated, washed with water and air-dried (yield: *ca.* 52% based on K_5_[SiW_11_O_39_Ru(H_2_O)]·10H_2_O). EA and ICP calcd (%) for C_40_H_60_N_8_O_54_SiW_11_RuCu_3_K: C 12.32, H 1.55, N 2.87, Cu 4.84, W 51.92, Ru 2.61; found: C 12.48, H 1.57, N 2.83, Cu 4.91, W 52.32, Ru 2.51. IR (KBr pellet; cm^–1^): 3395 (br, s), 1865 (w), 1608 (s), 1413 (m), 1219 (w), 1068 (w), 1008 (w), 957 (m), 913 (s), 881 (m), 788 (s).

### Synthesis of CR–**BPY**2

The CR–**BPY**2 was synthesized by a diffusion method in a test tube. A mixture of acetonitrile and water (1 : 1, 10.0 mL) was gently layered on the top of a solution of K_5_[SiW_11_O_39_Ru(H_2_O)]·10H_2_O (70.0 mg, 0.02 mmol) and Cu(NO_3_)_2_·3H_2_O (9.1 mg, 0.04 mmol) in water (5 mL). A solution of 4,4′-bipyridine (12.5 mg, 0.08 mmol) in acetonitrile (5 mL) was added carefully as the third layer. Brownish black block single crystals were separated after four weeks, washed with acetonitrile and water, and dried in air (yield: *ca.* 59% based on K_5_[SiW_11_O_39_Ru(H_2_O)]·10H_2_O). EA and ICP calcd (%) for C_40_H_58_N_8_O_52_SiW_11_RuCu_2_K: C 12.63, H 1.54, N 2.95, Cu 3.31, W 53.24, Ru 2.68; found: C 12.88, H 1.69, N 2.89, Cu 3.39, W 53.41, Ru 2.58. IR (KBr pellet; cm^–1^): 3404 (br, s), 1610 (s), 1415 (m), 1220 (w), 1075 (w), 1005 (w), 955 (m), 911 (s), 872 (m), 784 (s).

### X-ray crystallography

Data of CR–**BPY**1 and CR–**BPY**2 were collected on a Bruker Smart APEX CCD diffractometer with graphite-monochromated Mo-K*α* (*λ* = 0.71073 Å) using the SMART and SAINT programs.^34^ Their structures were determined and the heavy atoms were found by direct methods using the SHELXTL-97 program package.^35^ Crystallographic data for them are summarized in Table 3. Except some partly occupied solvent water molecules, the other non-hydrogen atoms were refined anisotropically. Hydrogen atoms within the ligand backbones were fixed geometrically at their positions and allowed to ride on the parent atoms. In both of the two structures, the ruthenium atoms were disordered in the equivalent positions of tungsten atoms. For CR–**BPY**2, several bond distances constraints were used to help the refinement on the **BPY** moiety, and thermal parameters on adjacent oxygen atoms of the polyoxometalate anion were restrained to be similar.

**Table 3:** Crystallographic data structure refinement for compounds CR–**BPY**1 and CR–**BPY**2

	CR–**BPY**1	CR–**BPY**2
Empirical formula	C_40_H_60_N_8_O_54_ SiW_11_RuCu_3_K	C_40_H_58_N_8_O_52_ SiW_11_RuCu_2_K
*M*, g mol^–1^	3898.19	3800.63
Crystal system	Tetragonal	Orthorhombic
Space group	*P*42_1_ *m*	*Pccn*
*a*, Å	24.415(3)	17.866(1)
*b*, Å	24.415(3)	22.192(2)
*c*, Å	15.337(4)	22.284(2)
*V*, Å^3^	9142(3)	8835.3(10)
*Z*	4	4
*D*_calcd_, g cm^–3^	2.777	2.857
*T*, K	296(2)	296(2)
Refl. collected/unique	61 657/10 865 *R*_int_ = 0.1270	42 658/7765 *R*_int_ = 0.0688
*μ*, mm^–1^	14.762	15.044
GOOF	0.972	1.022
*R*_1_ ^*a*^ (*I* > 2*σ*(*I*))	0.0509	0.0753
w*R*_2_ ^*b*^ (*I* > 2*σ*(*I*))	0.1256	0.1918
*R*_1_ ^*a*^ (all data)	0.1156	0.0971
w*R*_2_ ^*b*^ (all data)	0.1708	0.2042
Diff peak and hole, e Å^–3^	2.937/–1.494	5.265/–4.627

^*a*^ *R*_1_ = ∑||*F*_o_| – |*F*_c_||/∑|*F*_o_|.

^*b*^w*R*_2_ = [∑*w*(|*F*_o_|^2^ – |*F*_c_|^2^)/∑*w*(*F*_o_^2^)^2^]^1/2^; *w* = 1/[*σ* ^2^(*F*_o_^2^) + (*xP*)^2^ + *yP*], *P* = (*F*_o_^2^ + 2*F*_c_^2^)/3, where *x* = 0.1000, *y* = 0.0000 for CR–**BPY**1; *x* = 0.0874, *y* = 678.0471 for CR–**BPY**2.
