# Supplementary material for: Merging of the photocatalysis and copper catalysis in metal–organic frameworks for oxidative C–C bond formation
Source: Chem Sci. 2014 Oct 30;6(2):1035–42. doi: 10.1039/c4sc02362e (PMC5811127; doi:10.1039/c4sc02362e)
Supplement: Supplementary file 1 [file SC-006-C4SC02362E-s001.pdf]

Electronic Supplementary Material (ESI) for Chemical Science.
This journal is © The Royal Society of Chemistry 2014

Electronic Supplementary Information

Merging of the Photocatalysis and Copper Catalysis in Metal-Organic Frameworks for Oxidative C–C Bond Formation

Dongying Shi, Cheng He, Bo Qi, Cong Chen, Jingyang Niu and Chunying Duan

Contents

Section 1 Experimental Section

Section 2 Supplementary Structural Figures

Section 3 Characterizations of Catalysts

Section 4 Catalysis Details

Section 5 References

Section 1 Experimental Section

Fig. S1 Left Picture: TG curves of CR–**BPY**1 (brown line) and CR–**BPY**2 (blue line) in the flowing N_2 atmosphere, respectively. Right One: Solid state UV-Vis absorption spectra of CR–**BPY**1 (brown line) and $K_5[SiW_{11}O_{39}Ru(H_2O)]\cdot 10H_2O$ (blue line), respectively.

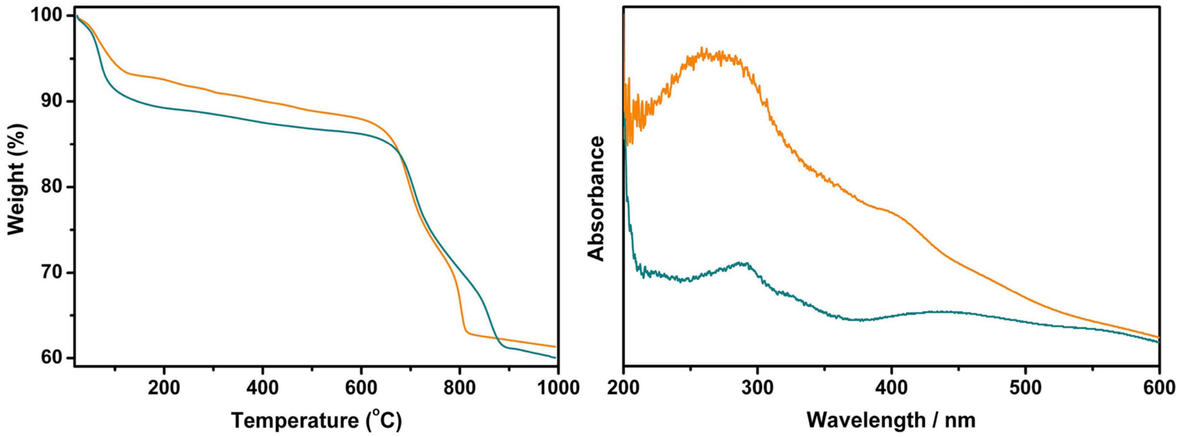

Fig. S2 Left Picture: Solid state cyclic voltammetry of $Cu(NO_3)_2{\cdot}3H_2O$; Right One: Solid state cyclic voltammetry of $K_5[SiW_{11}O_{39}Ru(H_2O)]{\cdot}10H_2O$; Below Pictures (a–c): Solid state cyclic voltammetry of CR–**BPY**2 repeated; Scan rate: 50 mV·s^{-1}.

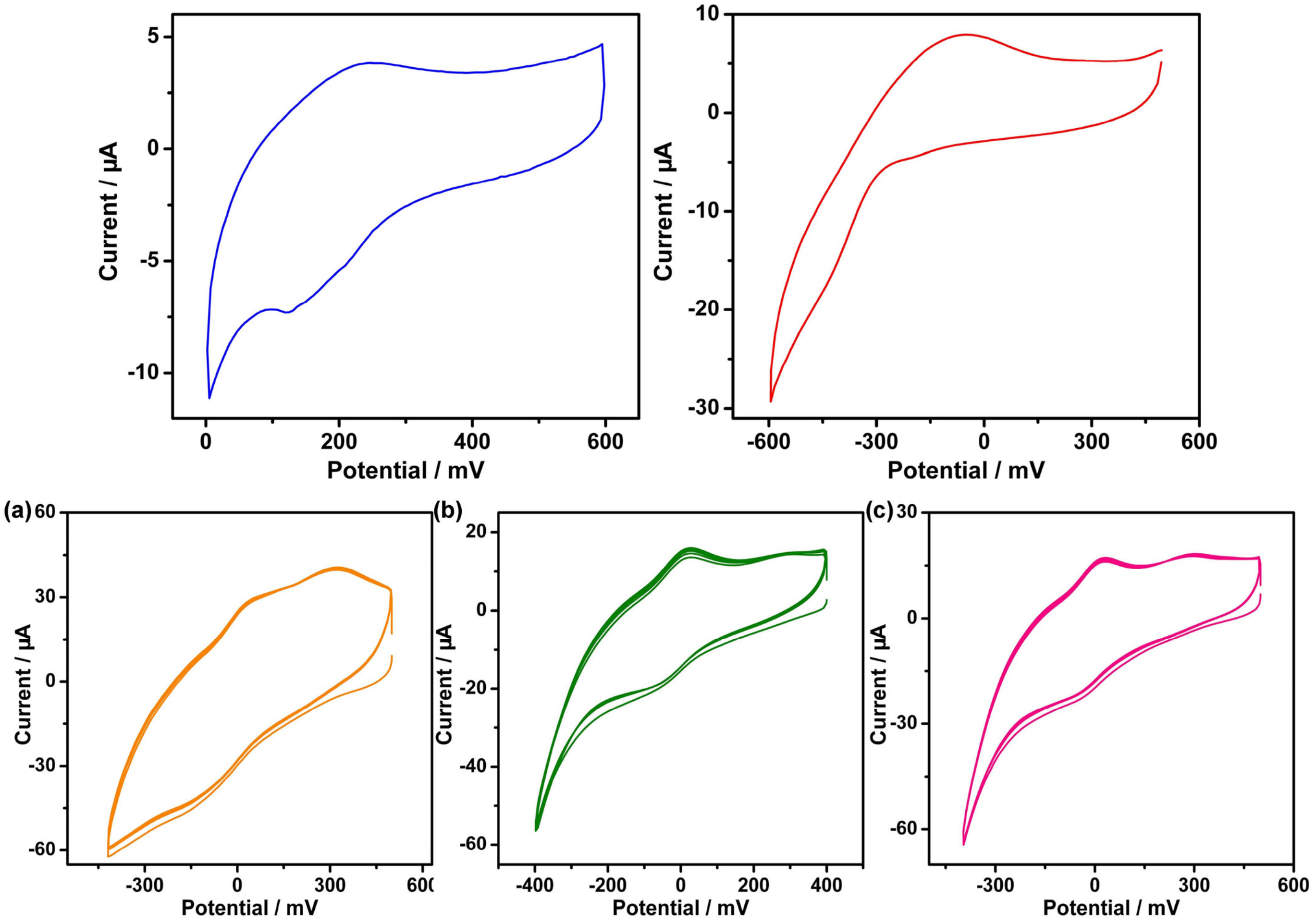

Section 2 Supplementary Structural Figures

Fig. S3 Chemical structure of CR–**BPY**1 with selected numbering scheme, showing the linkage of the polyoxometalate between the two sheets. Hydrogen atoms and lattice water molecules are omitted for clarity.

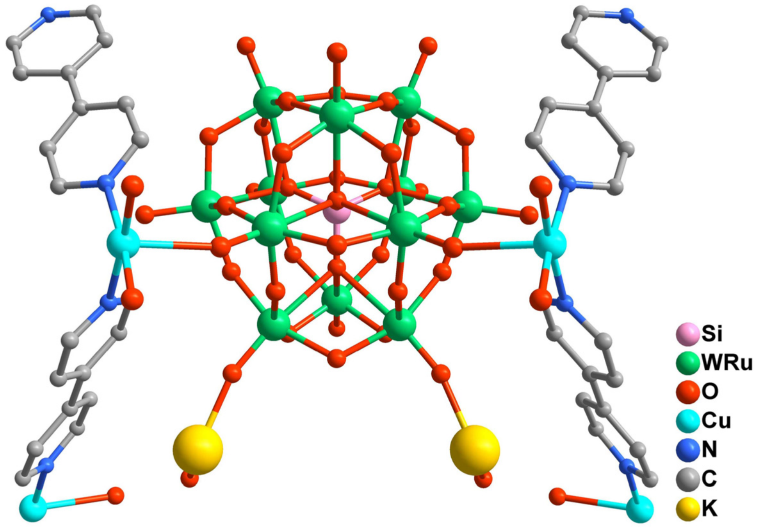

Fig. S4 The coordinate environment of Cu1(II) and Cu2(II) cations in CR–**BPY**1.

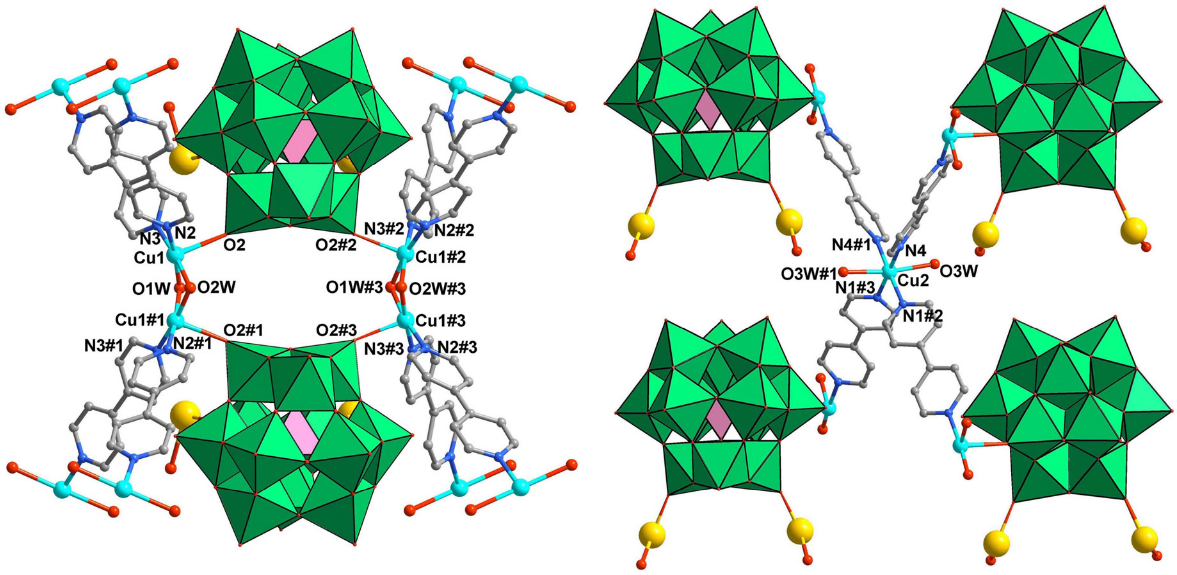

Selective bond distance (Å) in CR–**BPY**1 around Cu(1): Cu(1)–N(2) 1.984(12), Cu(1)–N(3) 1.997(10), Cu(1)–O(2) 2.366(11), Cu(1)–O(1W) 1.950(9), Cu(1)–O(2W) 1.973(9).

Selective bond distance (Å) in CR–**BPY**1 around Cu(2) (Symmetry code: #1 $-x$, $-y$, z; #2 x, y, $1 + z$; #3 $-x$, $-y$, $1 + z$): Cu(2)–N(4) 2.062(10), Cu(2)–N(1)$^{\#2}$ 2.060(11), Cu(2)–O(3W) 2.397(11).

Fig. S5 The 2D wavy-like Cu–**BPY** sheet of CR–**BPY**1, which was connected by **BPY** and μ_2-water bridges as well as Cu(II) connectors.

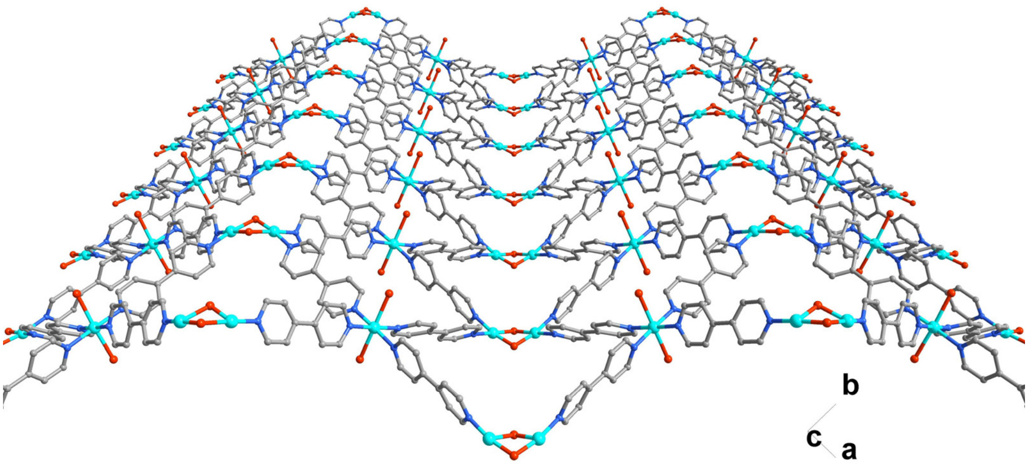

Fig. S6 View of the crystal packing of CR–**BPY**1 along the *a* direction.

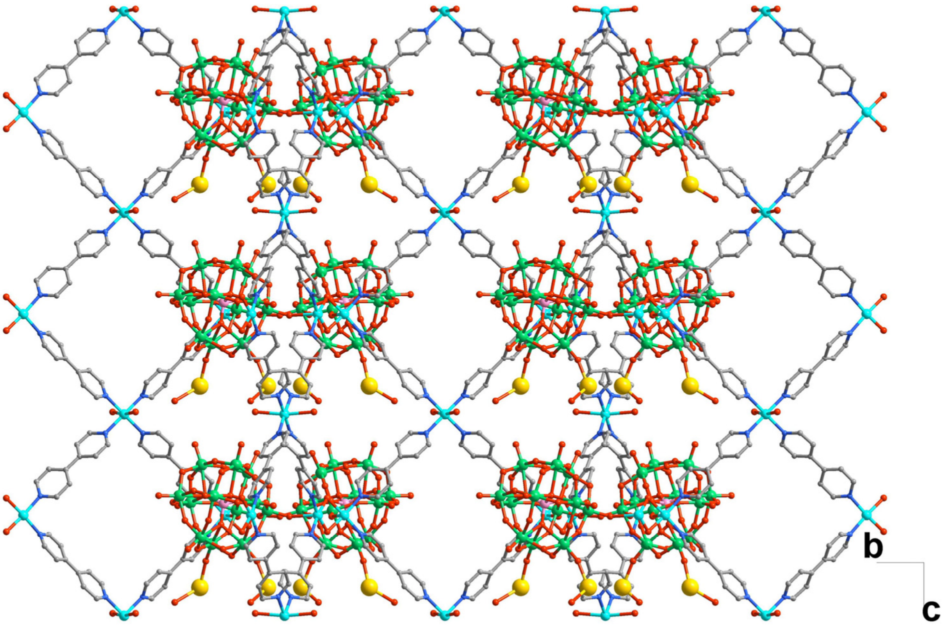

Fig. S7 The coordinate environment of Cu1(II) and Cu2(II) cations in CR–**BPY**2.

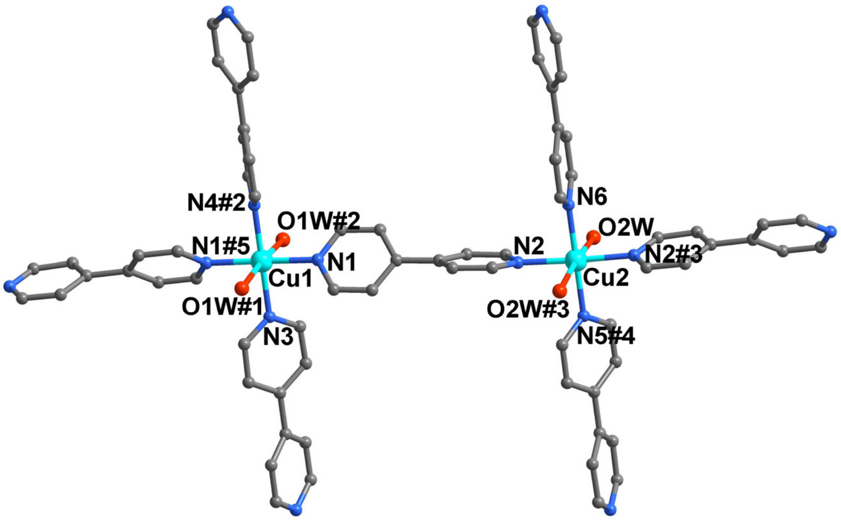

Selective bond distance (Å) in CR–**BPY**2 (Symmetry code: #1 *x*, –0.5 – *y*, 0.5 + *z*; #2 –0.5 – *x*, *y*, 0.5 + *z*; #3 –0.5 – *x*, 0.5 – *y*, *z*; #4 –0.5 – *x*, *y*, –0.5 + *z*; #5 –0.5 – *x*, –0.5 – *y*, *z*): Cu(1)–N(1) 2.019(4), Cu(1)–N(3) 2.040(6), Cu(1)–N(4)$^{\#2}$ 2.038(6), Cu(1)–O(1W)$^{\#1}$ 2.478(8), Cu(2)–N(2) 2.046(5), Cu(2)–N(6) 2.018(7), Cu(2)–N(5)$^{\#4}$ 2.036(7), Cu(2)–O(2W) 2.759(9).

Fig. S8 Crystal structure of CR–**BPY**2 showing the stacking pattern of the grid-like sheets with polyoxoanions $[SiW_{11}O_{39}Ru(H_2O)]^{5-}$ imbedded.

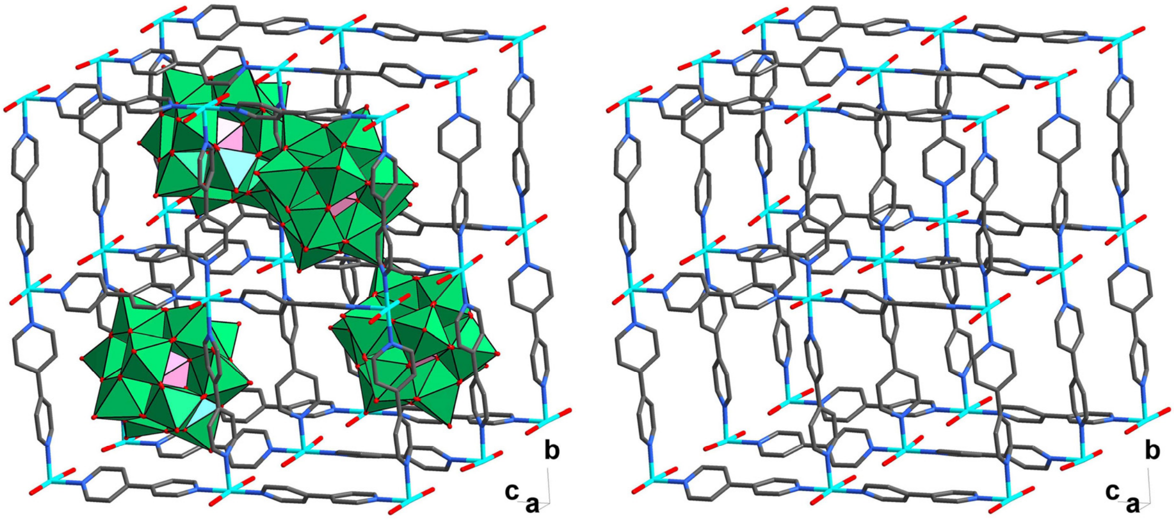

Section 3 Characterizations of Catalysts

Typical Procedure for Substrate Penetration of the MOF:

Substrate Penetration: Before the adsorption of substrate, CR–**BPY**1 was first briefly air-dried on a filter paper and fully dried out the guest water molecules in a vacuum oven (100 °C, 12 hours). Substrate penetration studies were carried out by soaking CR–**BPY**1 in 2.0 mL 1,4-dioxane solution containing 0.50 mmol acetophenone or 0.25 mmol *N*-phenyl-tetrahydroisoquinoline for 24 hours. After the soaked CR–**BPY**1 was rapidly washed with 1,4-dioxane solution several times to remove the substrate molecules adsorbed on the external surfaces of the crystals.

Fig. S9 ^{1}H NMR of CR–**BPY**1 in DMSO-d$_6$/DCl: CR–**BPY**1 (a), that after being penetrated with acetophenone (b), and that after being penetrated with *N*-phenyl-tetrahydroisoquinoline (c).

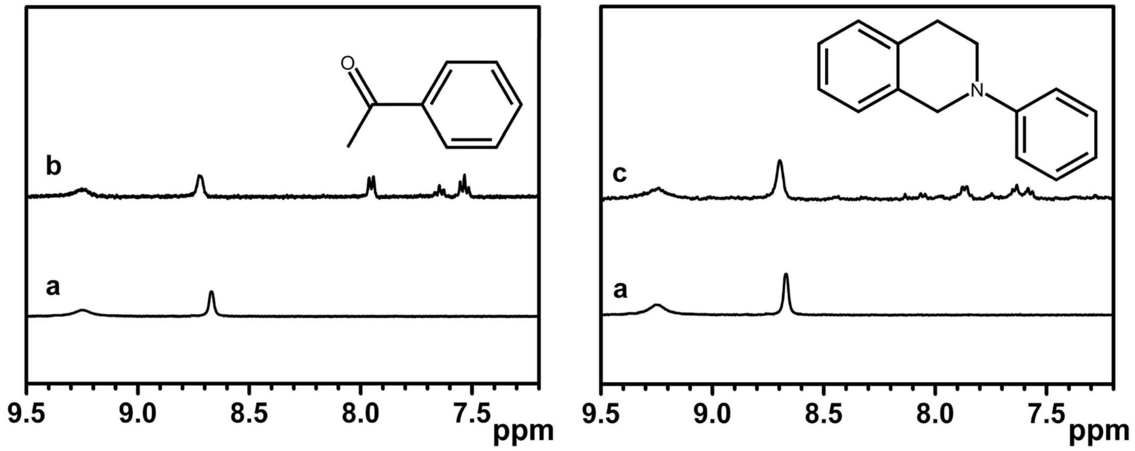

Fig. S10 Left Picture: Solid state IR spectra of acetophenone (a), CR–**BPY**1 (b), and solids of CR–**BPY**1 obtained after the absorption of acetophenone (c). Right One: Solid state IR spectra of *N*-phenyl-tetrahydroisoquinoline (a), CR–**BPY**1 (b), and solids of CR–**BPY**1 obtained after the absorption of *N*-phenyl-tetrahydroisoquinoline (c).

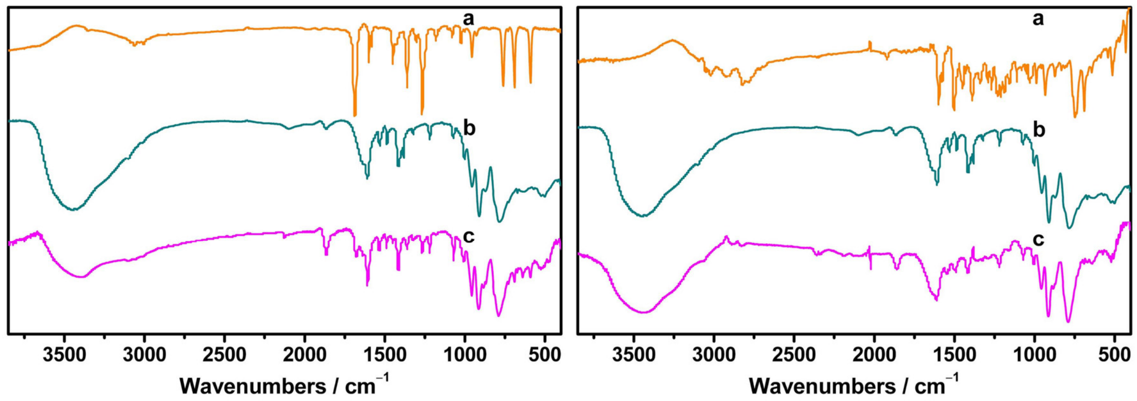

Typical Procedure for Dye Uptake of the MOF:

Dye Uptake: Before the adsorption of dye, CR–**BPY**1 was first briefly air-dried on a filter paper and fully dried out the guest water molecules in a vacuum oven (100 ^{o}C, 12 hours). UV-Vis measurement of 2′,7′-dichlorofluorescein dye released from dried CR–**BPY**1 (7.6 mg, 2 μmol) was soaked in a methanol solution of 2′,7′-dichlorofluorescein dye (24 mM, 2 mL) on a constant temperature oscillation incubator overnight. The resulting crystals were filtered and rapidly washed with methanol thoroughly till the solution become colorless (to remove any dye from the crystals' surfaces), and then dried under a stream of air. The dried samples were dissociated by concentrated hydrochloric acid, the resultant clear solution with light olivine color was diluted to 25 mL and adjusted to a pH of 0.5. Absorption experiment was performed on a UV-Vis spectrophotometer. The concentration of 2′,7′-dichlorofluorescein dye was determined by comparing the UV-Vis absorption with a standard curve.

Fig. S11 Left Picture: UV-Vis measurement of released 2′,7′-dichlorofluorescein dye released from CR–**BPY**1. Right One: The standard linear relationship between the absorption and the concentration of 2′,7′-dichlorofluorescein dye.

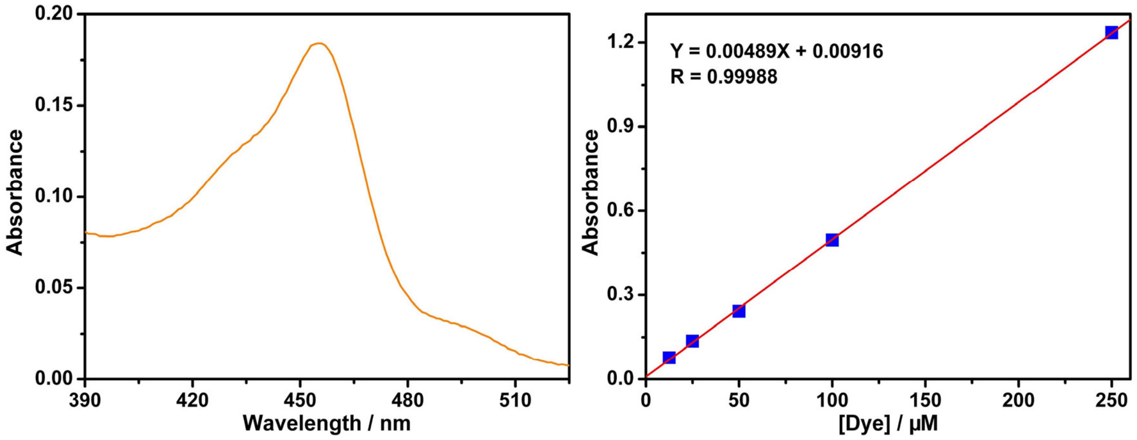

Fig. S12 Left Picture: Emission spectrum of *N*-phenyl-tetrahydroisoquinoline (0.1mM) in dichloromethane. Right One: Emission spectrum of $K_5[SiW_{11}O_{39}Ru(H_2O)]\cdot 10H_2O$ (0.1% in weight) upon addition of *N*-phenyl-tetrahydroisoquinoline (0.5 mM) in dichloromethane, excitation at 362 nm.

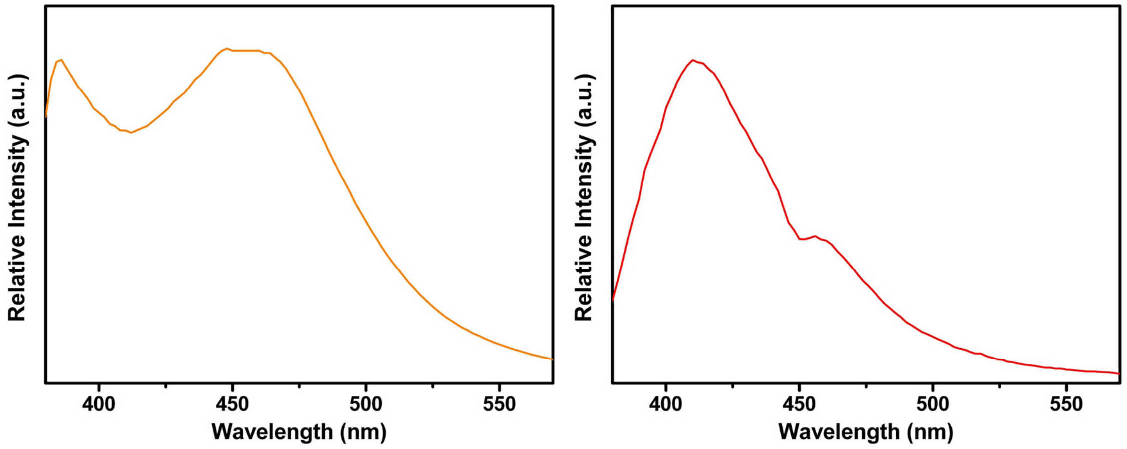

Fig. S13 Left Picture: The ESI-MS of the CH_2Cl_2 suspension containing *N*-phenyl-tetrahydroisoquinoline and CR–**BPY**1 before irradiation. Right One: The ESI-MS of the CH_2Cl_2 suspension containing *N*-phenyl-tetrahydroisoquinoline and CR–**BPY**1 after irradiation for 3 hours.

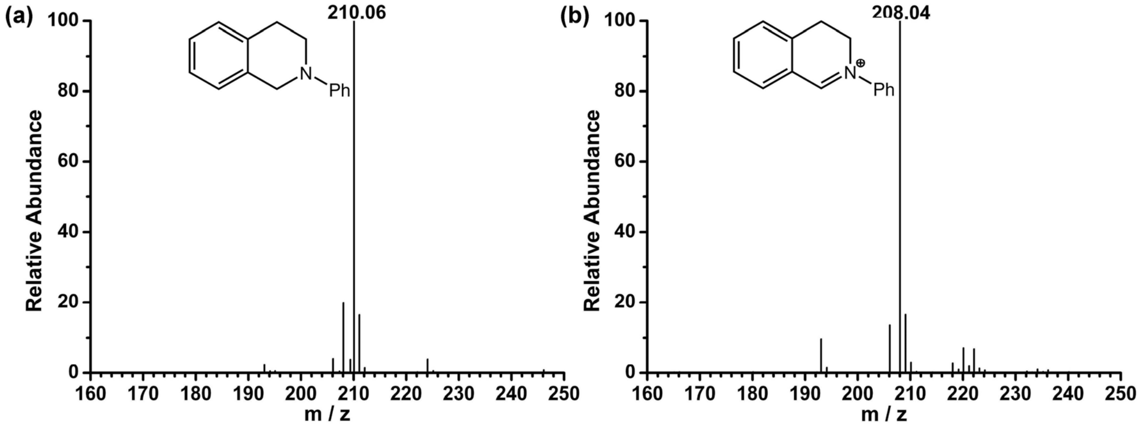

Section 4 Catalysis Details

Procedure for C–C coupling reactions of *N*-phenyl-tetrahydroisoquinoline and CH_3NO_2:

To a 10 mL quartz glass tube equipped with a magnetic stir bar was added *N*-phenyl-tetrahydroisoquinoline (52.3 mg, 0.25 mmol), catalyst (9.5 mg, 2.50 μmol) and CH_3NO_2 solvent (2.0 mL). The reaction was stirred under oxygen atmosphere at a distance of ~8 cm from a 18 W fluorescent lamp for 24 hours. After completion of the reaction, the resulting suspension was centrifuged. Removal of the solvent in vacuo yielded *N*-phenyl-tetrahydroisoquinoline derivative. The conversions were directly determined by ^1H NMR analysis of the reaction solution.

Fig. S14 Powder XRD pattern of CR–**BPY**1 (Left Picture) and CR–**BPY**2 (Right One) showing the calculated pattern based on the single-crystal solution and after three runs of the C–C coupling reaction (a-Simulated, b-Experimental, c-Recovery catalyst after three times runs).

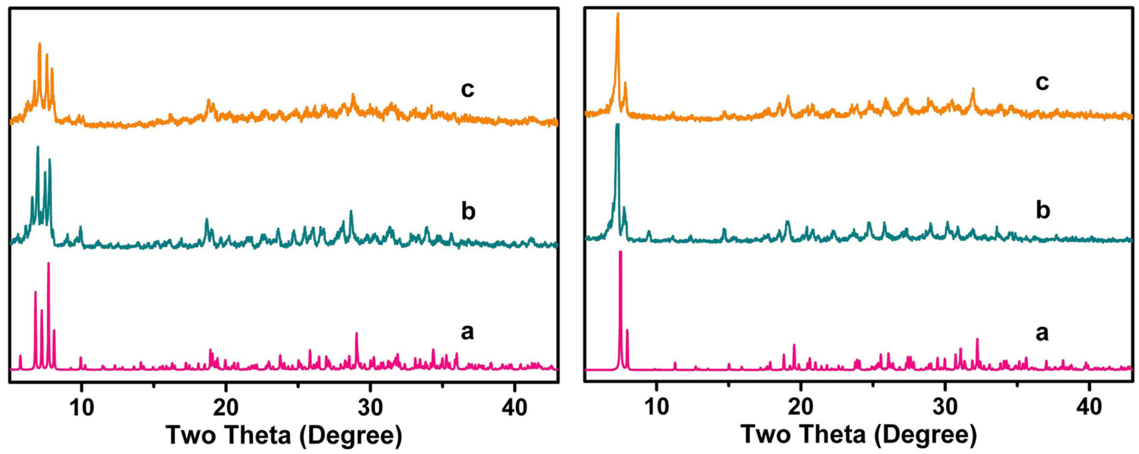

Fig. S15 SEM image of the initial crystals CR–**BPY**1 with size about 0.1 mm, with the catalytic yield of 94% in 36 hours (a); and the fine crystalline materials with size 0.5~2.0 μm by grinding CR–**BPY**1 crystals, with the catalytic yield of 94% in 32 hours (b).

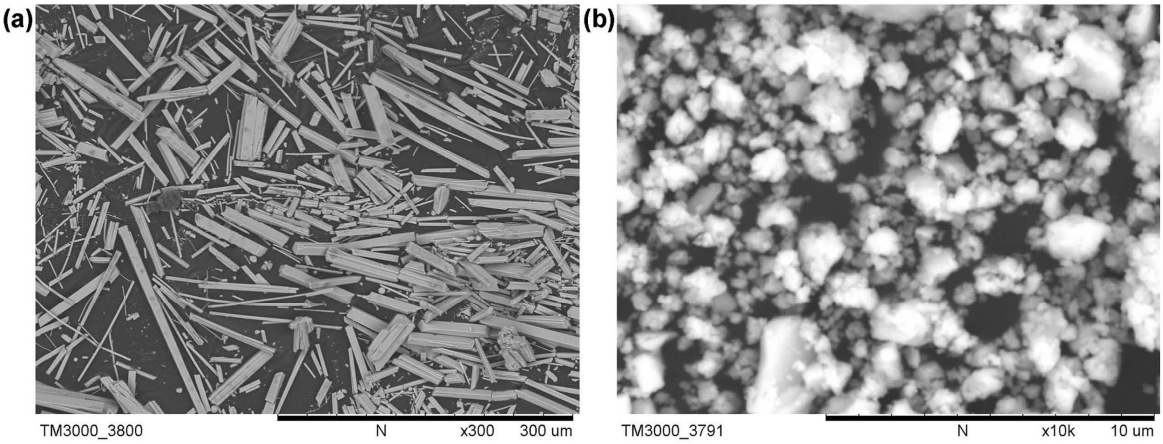

Procedure for C–C coupling reactions of *N*-phenyl-tetrahydroisoquinoline and acetophenones:

To a 10 mL quartz glass tube equipped with a magnetic stir bar was added *N*-phenyl-tetrahydroisoquinoline (52.3 mg, 0.25 mmol), CR–**BPY**1 (9.5 mg, 2.50 μmol), *L*-proline (5.8 mg, 0.05 mmol), acetophenone (58 μL, 0.50 mmol) and 1,4-dioxane solvent (2.0 mL). The reaction was stirred under oxygen atmosphere at a distance of ~8 cm from a 18 W fluorescent lamp for 60 hours. The reaction mixture was then centrifuged and evaporated under reduced pressure. The residue was purified by flash chromatography on alkalescence silica gel to afford corresponding products. The conversions were determined by ^{1}H NMR analysis of the reaction solution.

Table S1 Recycling of catalyst CR–**BPY**1 as well as the controlled trial for *N*-phenyl-tetrahydroisoquinoline and acetophenone reactions after irradiation of 60 hours.

Entry	Catalysts	Conversion (%)
**1**	CR–**BPY**1 (Round 1), *L*-proline, 18 W fluorescent lamp	72
**2**	CR–**BPY**1 (Round 2), *L*-proline, 18 W fluorescent lamp	68
**3**	CR–**BPY**1 (Round 3), *L*-proline, 18 W fluorescent lamp	65
**4**	$[SiW_{11}O_{39}Ru(H_2O)]^{5-}$, *L*-proline, 18 W fluorescent lamp	19
**5**	$[SiW_{11}O_{39}Ru(H_2O)]^{5-}$, *L*-proline, Cu^{2+}, 18 W fluorescent lamp	21
**6**	Cu^{2+}, *L*-proline, 18 W fluorescent lamp	25
**7**	$[SiW_{11}O_{39}Ru(H_2O)]^{5-}$, 18 W fluorescent lamp	<10
**8**	Cu^{2+}, 18 W fluorescent lamp	<10
**9**	no catalyst, 18 W fluorescent lamp	<10

Verification for the C–C coupling products:

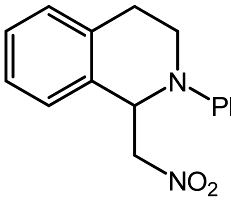

1-(nitromethyl)-2-phenyl-1,2,3,4-tetrahydroisoquinoline. ^{1}H NMR ($CDCl_3$): δ 7.40-7.12 (m, 6H); 6.99-6.97 (m, 2H); 6.86-6.83 (m, 1H); 5.57-5.53 (t, 1H); 4.90-4.85 (dd, 1H); 4.59-4.54 (dd, 1H); 3.70-3.55 (m, 2H); 3.13-2.99 (m, 1H); 2.82-2.76 (m, 1H).

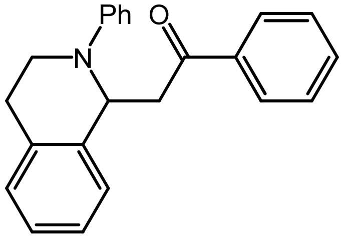

1-phenyl-2-(2-phenyl-1,2,3,4-tetrahydroisoquinolin-1-yl)ethanone (Entry 1, Table 2). ^{1}H NMR ($CDCl_3$): δ 7.98-7.82 (m, 2H); 7.58-7.50 (m, 1H); 7.46-7.36 (m, 2H); 7.26-7.08 (m, 6H); 7.03-6.95 (m, 2H); 6.79-6.71 (m, 1H); 5.71-5.62 (m, 1H); 3.66-3.36 (m, 4H); 3.18-3.06 (m, 1H); 3.02-2.86 (m, 1H).

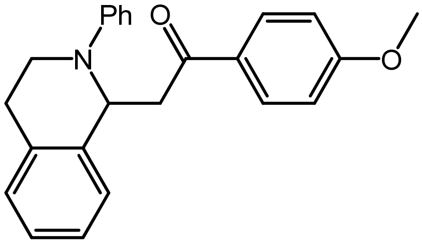

1-(4-methoxyphenyl)-2-(2-phenyl-1,2,3,4-tetrahydroisoquinolin-1-yl)ethanone (Entry 2, Table 2). ^{1}H NMR ($CDCl_3$): δ 7.93 (d, 2H); 7.83-7.03 (m, 7H); 6.93 (d, 2H); 6.75 (m, 2H); 5.66 (t, 1H); 3.86 (s, 3H); 3.71-3.65 (m, 2H); 3.55 (m, 1H); 3.34 (m, 1H); 3.11 (m, 1H); 2.92 (m, 1H).

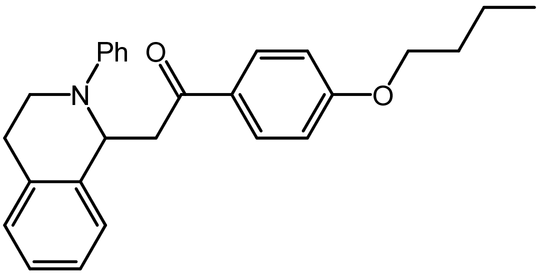

1-(4-butoxyphenyl)-2-(2-phenyl-1,2,3,4-tetrahydroisoquinolin-1-yl)ethanone (Entry 3, Table 2). ^{1}H NMR ($CDCl_3$): δ 7.93 (d, 2H); 7.83-7.03 (m, 7H); 6.93 (d, 2H); 6.75 (m, 2H); 5.66 (t, 1H); 3.86 (s, 3H); 3.71-3.65 (m, 2H); 3.55 (m, 1H); 3.34 (m, 1H); 3.11 (m, 1H); 2.92 (m, 1H).

Synthesis of 1-(3',5'-di-tert-butyl[1,1'-biphenyl]-4-yl)-ethanone:[S1]

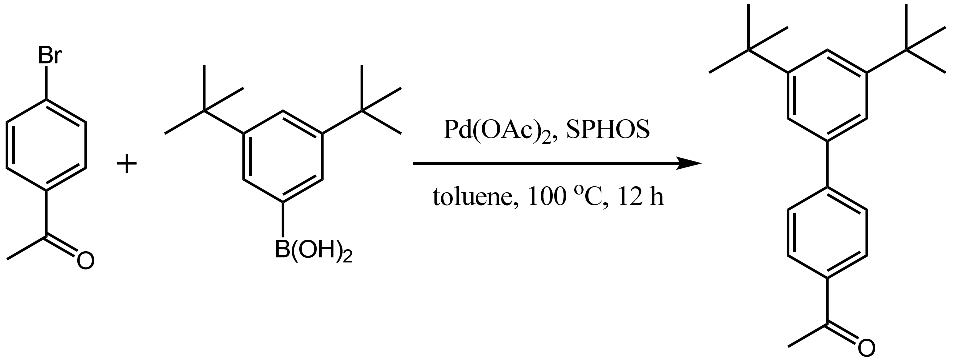

SPHOS[S2] (0.025 mmol, 10.3 mg), $Pd(OAc)_2$ (0.013 mmol, 2.8 mg), 3,5-di-tert-butylbenzeneboronic acid (1.88 mmol, 441 mg), and K_3PO_4 (2.51 mmol, 533 mg) were added to a flame-dried Schlenk flask. After evacuating and refilling with argon, toluene (4 mL) and 4-bromoacetophenone (1.25 mmol, 166 μL) were added via syringe. After stirring at 100 °C for 12 hours, the mixture was allowed to cool to room temperature, and was filtered through a plug of silica gel, eluting with diethyl ether. Volatiles were removed, and the residue was purified by flash chromatography, eluting with 10% ethyl acetate in hexanes. White solid was obtained (Yield: 71%). ^{1}H NMR ($CDCl_3$): δ 8.04 (2H, d, Ph); 7.69 (2H, d, Ph); 7.49 (1H, s, Ph); 7.44 (2H, d, Ph); 2.64 (3H, s, COC**H**$_3$); 1.39 (18H, s, 2 × C(C**H**$_3$)$_3$).

Section 5 References

S1. A. R. Chianese, A. Mo and D. Datta, *Organometallics*, 2009, **28**, 465–472.

S2. SPHOS = 2-dicyclohexylphosphino-2',6'-dimethoxy-1,1'-biphenyl.
